# Trypanosomiasis vector control in Africa and Latin America

**DOI:** 10.1186/1756-3305-1-24

**Published:** 2008-08-01

**Authors:** Chris J Schofield, John P Kabayo

**Affiliations:** 1 ECLAT Coordinator, LSHTM, London ,WC1E7HT, UK; 2 PATTEC Coordinator, African Union, PO Box 200032, Addis Ababa, Ethiopia

## Abstract

Vectors of trypanosomiasis – tsetse (Glossinidae) in Africa, kissing-bugs (Triatominae) in Latin America – are very different insects but share demographic characteristics that render them highly vulnerable to available control methods. For both, the main operational problems relate to re-invasion of treated areas, and the solution seems to be in very large-scale interventions covering biologically-relevant areas rather than adhering to administrative boundaries. In this review we present the underlying rationale, operational background and progress of the various trypanosomiasis vector control initiatives active in both continents.

## Background

**Figure 1:**
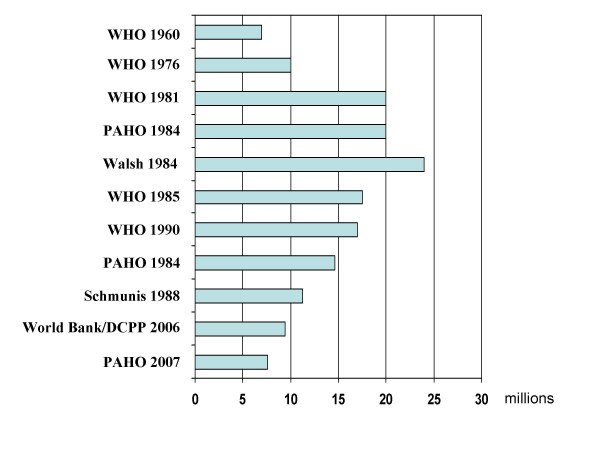
**The rise and fall of Chagas disease**. Estimates of Chagas disease prevalence 1960–2007.

The trypanosomiases are amongst the most serious of the so-called 'neglected tropical diseases' [1]. In the 1980s, American trypanosomiasis (Chagas disease, due to *Trypanosoma cruzi*) was believed to infect over 24 million people [2] with another 100 million considered at risk, although these estimates have since been reduced (Fig. 1). Chagas disease can be fatal in its early acute stage, but more usually progresses to a debilitating chronic phase – affecting up to 30% of those infected – involving severe cardiac lesions and, with some strains, intestinal lesions resulting in the so-called 'mega-syndrome' of severe intestinal dilations. Because of these debilitating effects, the World Bank [3] ranked Chagas disease as by far the most serious of the parasitic diseases affecting Latin America, with a social and economic impact far in excess of even the combined impact of other diseases such as malaria, leishmaniasis and schistosomiasis. By contrast, African trypanosomiasis (Sleeping Sickness) is invariably fatal if untreated, with the gambiense form generally causing a chronic disease leading to fatal sequelae after some years, and the more acute rhodesiense form often leading to death in just a few months. But the suite of closely-related animal trypanosomiases transmitted by tsetse in Africa (including *T. b. brucei, T. vivax, T. congolense*, and *T. simiae*) have an even greater impact, denying livestock over vast areas, and affecting agricultural production both directly and indirectly by limiting the use of draught animals for transport and ploughing. The World Health Organization currently estimates some 300,000 cases of human Sleeping Sickness [4], while the economic cost of animal trypanosomiasis in Africa has been estimated at US$4.75 billion per year [5].

American and African trypanosomiases are difficult to treat, and vaccines are unavailable. Two drugs – benznidazole and nifurtimox – can be used to treat Chagas disease, but they are currently only used in the acute stage or early chronic stage of infection, and both can cause severe side effects, sometimes life-threatening. Nifurtimox can also be used to treat gambiense Sleeping Sickness, although a range of other drugs is more widely used (Table 1). None is ideal, and the most widely used – melarsoprol (sometimes described as "a mixture of arsenic and antifreeze") – tends to be lethal for up to 10% of those treated. Other drugs are used to treat livestock, but tend to be expensive for African farmers, and show increasing problems of drug-resistance and drug-counterfeiting [6]. In both continents therefore, vector control is of crucial importance in trypanosomiasis control.

**Table 1:** Currently available treatments for human trypanosomiasis

	***Date of*** *** introduction***	***Days of treatment***						***Likelihood of *** ***serious side*** *** effects***
		***T. cruzi***		***gambiense***		***rhodesiense***		
		**early**	**late**	**early**	**late**	**early**	**late**	

Suramine	1916	-	-	-	-	40^a^	-	++
Pentamidine	1940	-	-	7	-	-	-	+/-
Melarsoprol	1949	-	-	-	10–30^b^	-	10–30^b^	+++++
Nifurtimox	1967	30–60	-	-	15–30^c^	-	-	++
Benznidazole	1972	30–60	-	-	-	-	-	++
Eflornithine	1990	-	-	-	7–14^d^	-	-	++

^a ^Suramine: 1 injection per week for 5 weeks

^b ^Melarsoprol: new regime being developed of 1 daily injection for 10 days

^c ^Nifurtimox: not yet registered for use against gambiense, but is being used on compassionate grounds when other treatments fail

^d ^Eflornithine: 4 intravenous perfusions per day

The vectors of these trypanosomiases – Triatominae (kissing bugs) in Latin America, Glossinidae (tsetse-flies) in Africa – are very different insects, but share certain characteristics that render them highly vulnerable to available vector control methods. Both are slowly-reproducing – often described as *K*-strategists in the terminology of MacArthur & Wilson [7] – and so have a limited capacity to repopulate areas where their abundance has been reduced. Moreover, their demographic strategies tend to lead to reduced genetic variability within each vector population, limiting their capacity to respond to vector control interventions through selection of new attributes such as insecticide-resistance [8]. No tsetse population has yet been selected for insecticide resistance, and amongst Triatominae only one focus of limited resistance was detected in *Rhodnius prolixus *in Venezuela during the 1970s, with some pyrethroid resistance now also being found amongst some *Triatoma infestans *populations in southern Bolivia and northwestern Argentina [9,10].

### Control of American trypanosomiasis (Chagas disease)

Chagas disease was first described by Brasilian clinician Carlos Chagas in 1909. Remarkably, Chagas not only described the causative agent, *Trypanosoma cruzi*, with most of its life-cycle and clinical features, he also described its vectors and some small animal reservoirs, and was amongst the pioneers – with José Pellegrino and Emmanuel Dias – in attempts to control it. The parasite, and vector species of Triatominae, are widely distributed in the Americas from the Great Lakes of northern USA to southern Argentina, generally in a silvatic cycle of transmission involving small nest-building animals such as opossums, armadillos, and various species of rodent. In much of Latin America however, species of Triatominae have adapted to live in the cracks and crevices of rural dwellings, emerging at night to feed on the sleeping occupants. While feeding, they may defaecate the remains of their previous bloodmeal, so that if infected, the parasites in the bug faeces can pass to the human host – especially across the mucosa of eye, mouth, or nose. As Carlos Chagas observed, since the bugs live in the cracks of poor-quality rural homes, then improving the houses should make them less suitable for the bugs, and house improvement programmes remain an important component of Chagas disease control in many areas. By the 1940s, attempts were also being made to eliminate domestic Triatominae by spraying kerosene over house walls, and even using military flame-throwers. But synthetic insecticides proved more effective (and less hazardous). Organochlorines such as BHC (lindane) and dieldrin were widely used until being progressively replaced by synthetic pyrethroids during the 1980s.

Venezuela in the 1960s [11] and also the Brasilian state of São Paulo [12], had shown that it was feasible to eliminate domestic Triatominae from rural houses by a consistent and widespread campaign of indoor insecticide spraying. This experience, coupled with pilot control trials in many other areas, laid the basis for the Brasilian national Chagas disease control programme, launched in 1983 with the declared objective of eradicating the main domestic vector, *Triatoma infestans*, which appeared to have no silvatic foci in Brasil. This campaign was highly successful. By 1986, *T. infestans *had been eliminated from almost 80% of its previously-known distribution in Brasil [13] but the programme was then halted due to outbreaks of dengue in the main coastal cities of Brasil – the 600 or so field staff of the rural Chagas control programme were abruptly withdrawn and sent to hunt *Aedes aegypti *in the urban areas, and the Chagas control programme collapsed.

This Brasilian campaign showed two important lessons. Firstly, the methods and operational strategy appeared adequate, such that there seemed no technical impediment to eliminating domestic Triatominae on a large-scale. But secondly, achieving this objective would be impossible if similar interventions were not also being carried out in neighbouring countries (*T. infestans *is no respecter of national boundaries), and would require a continued political commitment to maintain the campaign until its objectives had been reached. The enlightened response was the Southern Cone Initiative, a multinational programme designed to eliminate domestic *T. infestans *throughout its known distribution not just in Brasil, but also in Argentina, Bolivia, Chile, Paraguay, Uruguay, and southern Peru [14]. Approved by Ministers of Health in 1991 (Resolution 04-3-CS) and coordinated by the Pan American Health Organization (PAHO), the work of the Southern Cone Initiative began in 1992, with vector control interventions progressively covering the whole distribution of *T. infestans *– an area estimated at just over 6 million Km^2^. At the time of writing, the distribution of *T. infestans *appears to have been reduced to under 1 million Km^2^, with Chagas disease transmission formally declared to have been halted in Uruguay, Chile, most of Brasil, and substantial areas of Paraguay and Argentina. Domestic transmission has also been substantially reduced in Bolivia and southern Peru, although it remains high in parts of the Chaco region of southern Bolivia and northwestern Argentina [15,16].

The success of the Southern Cone Initiative helped to promote two similar multinational initiatives against Chagas disease – in Central America and the Andean Pact region – both launched in 1997, together with interstate initiatives in Mexico [17], and a large-scale Chagas disease surveillance initiative for the countries of the Amazon basin launched in 2002 [18]. The Central American Initiative (Guatemala, Honduras, El Salvador, Belize, Nicaragua, Costa Rica, Panama) is mainly targeted at the elimination of *Rhodnius prolixus *(which is exclusively domestic in Central America), elimination of domestic colonies of *T. dimidiata *(which has widespread silvatic ecotopes and cannot be targeted for complete area-wide elimination), and also control of *R. pallescens *in Panama. At the time of writing, *R. prolixus *appears to have been eliminated from almost all of its previously-known distribution in Central America, and house infestation rates with *T. dimidiata *have been substantially reduced in all countries of the region except Costa Rica [19]. The Andean Pact Initiative (Colombia, Venezuela, Ecuador, Peru) has similar objectives, but has been less successful, with only limited vector control interventions being carried out.

Meanwhile, on the other side of the Atlantic, the initiatives against American trypanosomiasis were being carefully analysed by those involved with large-scale control of the African trypanosomiases. In particular, the operational experience with Chagas disease control seemed to show the importance of an area-wide approach (rather than smaller projects of limited scope and duration), and also the importance of a clear political mandate for multinational interventions.

### Control of African trypanosomiasis (Sleeping Sickness and Nagana)

Attempts to control African trypanosomiasis have a long history, dating from the colonial period when the European powers were concerned by epidemics of the human disease and the chronic loss of livestock impeding both transport and agriculture [20]. Their attempts – based largely on active detection and treatment of sleeping sickness cases, combined with major programmes to control tsetse by bush-clearance (to eliminate tsetse resting sites), wild game culling (to reduce the parasite reservoirs and host availability for tsetse), and insecticidal spraying of tsetse resting sites – were largely successful. Sleeping Sickness case reports declined markedly from the 1920s to the 1960s, and considerable areas were cleared of tsetse – especially in parts of northern Nigeria.

**Figure 2:**
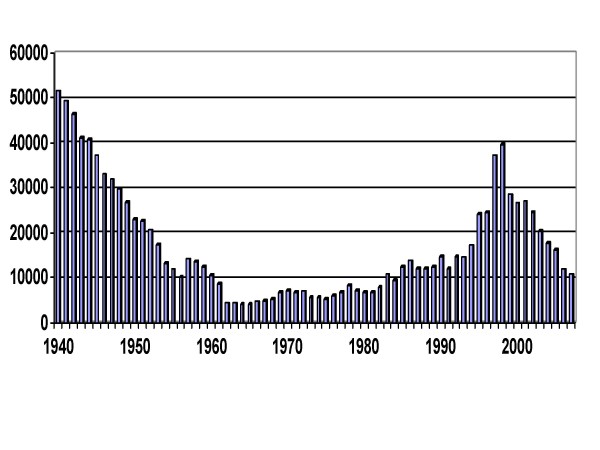
**The fall and rise (and fall) of African Sleeping Sickness**. Sleeping Sickness cases officially notified to WHO 1940–2007 (figures courtesy of J. Jannin & P. Simarro, WHO Geneva). Note that WHO estimates the real prevalence may be up to 12 times the reported figure.

But following the wars of independence during the 1960s, and the progressive withdrawal of colonial infrastructure, case incidence of Sleeping Sickness began to rise (Fig. 2) and routine tsetse control activities were interrupted in many of the endemic countries. An important initiative by the World Health Organization (WHO) led to steady improvements in the supply of drugs to treat Sleeping Sickness, such that by 1998 all such drugs could be offered free-of-charge and WHO – in partnership with industry – was able to offer increasing support for national medical services involved in active detection and treatment. Since 1998, overall case reports of human Sleeping Sickness have started to decline again (Fig. 2) although transmission rates remain high in parts of some countries – most notably southern Sudan, Democratic Republic of Congo (formerly Zaire), and northern Angola [4,21].

In parallel, a number of externally-financed projects to control tsetse were set up in countries of interest to external donor organisations – particularly in parts of francophone Africa (eg. [22-24]], the Regional Tsetse and Trypanosomiasis Control Programme of Zimbabwe, Zambia, Mozambique and Malawi [25] and the various FITCA projects (Farming in Tsetse Controlled Areas) supported by the European Commission. Such projects were in administratively-defined areas and were run for an administratively-specified time. They were generally successful whilst in operation, but generally proved unsustainable once the formal project reached its endpoint, so many were ultimately judged to be failures. Rather different was the tsetse eradication project on the island of Zanzibar sponsored by the International Atomic Energy Agency (IAEA). Although designed as a test-bed for tsetse control by large-scale release of laboratory-reared radiation-sterilised male tsetse (SIT) – and so proving rather costly – the project succeeded in complete elimination of tsetse (*Glossina austeni*) from the island [26] raising the question of whether or not similar tsetse elimination might be possible on mainland Africa.

Those involved were quick to realise the parallels with American trypanosomiasis control. In both Africa and in Latin America there had been a series of control projects following administratively-defined boundaries (rather than covering biologically-relevant areas) that had been technically successful until being halted before a sustainable end-point had been reached. The Latin American response came through multinational initiatives designed to promote political continuity of action and to cover the biologically relevant area represented by the entire distribution of the target vector species. In Latin America, the first step had been scientific debate [cf. [27]], followed by the political mandate (Ministerial resolution number 04-3-CS in 1991), designation of coordinating body (PAHO), and work with each country within the mandated framework.

For Africa therefore, the idea of a Pan-African initiative against tsetse and trypanosomiasis was discussed and recommended at the 25^th ^ISCTRC (International Scientific Council for Trypanosomiasis Research and Control) in Mombasa, Kenya, in October, 1999. The recommendation was presented to the 36^th ^summit of the OAU (Organization for African Unity, now African Union) in Lomé, Togo, in July 2000. In response, the Heads of State and Government of the 36 member states of the OAU passed resolution AHG/Dec.156 XXXVI recognising the seriousness of the tsetse and trypanosomiasis problem, and calling on member states "*to act collectively...... to render Africa tsetse-free within the shortest time possible*". With this mandate, the OAU set up the Pan African Tsetse and Trypanosomiasis Eradication Campaign (PATTEC), which is now an integral part of the AU Commission for Rural Development.

PATTEC was formally launched at the 26^th ^ISCTRC meeting in Ouagadougou in October 2001, and its plan of action endorsed by the OAU summits in Lusaka (OAU Decision AHG/Dec.169/XXXVII) and Durban (OAU/AU Decision CM/Dec.661/LXXVI.2002). The General Conferences of the FAO and IAEA adopted resolutions in support of the initiative (FAO 31^st ^Gen.Conf. Res 4.2001; IAEA 45^th ^Gen Conf. Res GC(45)/RES/12), as did the World Health Assembly of WHO (Res WHA56.7).

The PATTEC mandate encompasses the whole of sub-saharan Africa where tsetse are endemic, although it is well recognised that it may not be feasible or necessary to eradicate all tsetse species and populations, and that not all regions are currently amenable to intervention. The PATTEC vision is of long-term activities – extending perhaps over 30–50 years – with the hope that during this period each target area will present a suitable 'window of opportunity' when political and economic stability will permit large-scale interventions against tsetse and trypanosomiasis [28]. No specific technical package is defined, since elimination of each tsetse population will have its own technical requirements, and each target region has its own background, experience and practical capabilities. Thus each target area will derive its own technical package, drawing from the wide range of available control methods – including combinations of traps and insecticide-impregnated targets, insecticide-treated cattle, ultra-low dose aerial spraying (SAT) or ground-spraying or fogging of tsetse resting sites, and SIT if feasible and necessary for definitive elimination of the target population. Much depends on the ability to define biologically feasible targets – ie. tsetse populations that are sufficiently geographically discrete that reinvasion would be unlikely, or that occupy regions around which effective barriers could be maintained until neighbouring areas can be treated. To assist in this, PATTEC has set up a research network supported by the Leverhulme Trust (LTTRN) [29] that uses phenetic and genetic markers to assess geographical structuring of target tsetse populations [eg. [30,31]].

Although AU-PATTEC is still at an early stage, considerable progress has been made, most notably in reviving national programmes in a number of countries. Almost all the tsetse-endemic countries now have designated PATTEC focal points within the Ministry of Livestock and/or Ministry of Health, and a series of multinational interventions has begun with support from the national Governments. In addition, the African Development Bank has provided a series of loans and grants totalling some $72 million to support tsetse elimination activities in Ethiopia, Kenya, Uganda, Mali, Burkina Faso, and Ghana, with several other countries currently negotiating similar arrangements. The new regional programmes include the following:

- The 'Cotton Belt' of Mali, Burkina Faso, and northern Ghana (main targets: *G.p. gambiensis, G.m. submorsitans, G. tachinoides*) which is planned to extend progressively also into Cote d'Ivoire, Guinea, and Senegal

- The Lake Victoria Basin, including parts of Kenya, Uganda, Tanzania, and Ruanda (main targets: *G.f. fuscipes, G.m. submorsitans, G. pallidipes*)

- Southern Rift Valley – southern Ethiopia and neighbouring parts of Sudan (main target: *G. pallidipes*)

- The southeastern tsetse pocket (*G. brevipalpis, G. austeni*) of southern Mozambique and northeastern South Africa (KwaZulu Natal)

- The southern tsetse belt (*G.m. centralis*) of Botswana, Namibia, western Zambia and southern Angola.

Of these, the southern programme is currently the most advanced, and shows a clear example of what can be achieved through multinational cooperation. The southern *G.m. centralis *belt is extensive – over 40,000 km^2 ^– but relatively isolated from other tsetse populations. Control operations had been in progress in northern Botswana and western Zambia since the 1960s [20,32-34] but flies were still abundant in many areas [35]. In 2000, the Government of Botswana initiated a new campaign designed to eliminate *G.m. centralis *from the Okavango delta, expecting then to deploy a series of trap barriers along the frontiers to protect against reinvasion from neighbouring countries. The control interventions, based on sequential aerial spraying (SAT) and traps for monitoring tsetse densities, appear to have been very successful, with no tsetse encountered since completion of the interventions in June 2006 [36]. But the strategic approach has changed. Instead of deploying barriers against reinvasion, the control programmes have now extended across the tsetse infested Caprivi strip of Namibia, into southern Angola and western Zambia, with the aim of eliminating the entire *G.m. centralis *belt of the four countries [37,38]. The programme is being almost entirely financed by the Governments involved, with a high degree of cross-border cooperation, including cross-border staff deployment.

## Conclusion

The human trypanosomiases – Chagas disease in Latin America, Sleeping Sickness in Africa – may be eliminated as major public health problems within the next decade or so. In Latin America, the campaigns have focused primarily on vector control – elimination of domestic vector populations by indoor insecticide spraying – although the strategy is now changing to give additional emphasis to detection and treatment of new cases that may occur as a result of adventitious silvatic Triatominae entering houses [16]. In Africa, Sleeping Sickness control relies primarily on case detection and treatment, although it is increasingly recognised that operational advances will more likely be sustained where vector control is also carried out effectively. But for the African animal trypanosomiases, that contribute so much to poverty, underdevelopment, and food insecurity, tsetse elimination is now seen as the primary approach for the long-term. And in both continents – Latin America and Africa – accumulating experience is showing that large-scale elimination of vector populations is feasible, and more likely to be sustainable in the face of changing circumstances and priorities.

Comparing the two situations, both before and after the current initiatives, may be premature in the light of on-going interventions, but seems to reveal a series of key features that may be conducive to success. Of paramount importance is the political mandate, giving legitimacy to large-scale interventions, and greatly assisting – although by no means guaranteeing – the continuity of action required. This has been relatively weak for the Latin American initiatives, and requires constant reaffirmation as national policies change. It is stronger for the PATTEC initiative, and the mandated requirement for annual reporting to the AU Heads of State and Government provides regular opportunities for reaffirmation in the light of progress achieved. The political mandate also influences resource allocation, both in operational funds and the executive personnel who implement the control interventions. It is noteworthy that – so far – almost all funding for the current trypanosomiasis control initiatives, both in Latin America and in Africa, has come from national governments – either from national budgets or repayable loans.

Of parallel importance is the academic and research community. Where this is strong in some countries of Latin America, it has greatly helped to promote continuity of control interventions, as well as assisting in problem-solving and in programme monitoring and evaluation. Where it is weak however, or disconnected from the control programmes, it can be a distraction and hindrance [39]. The indication is that academia should be prepared to work closely with the vector control services, such that their activities will be mutually complementary, but with both working to ensure continuity of intervention and effective monitoring of progress. Of particular importance is that the research responds to the practical needs of the control programmes, rather than pursuing untried – and generally unneeded – technical innovations.

The importance of the political mandate and of the academic community is perhaps equally relevant for the control of any vector-borne disease, but for the trypanosomiases one further factor is of crucial importance. For both African and American trypanosomiasis, the vectors are exquisitely vulnerable to currently available control techniques. The low reproductive rates and limited genetic variability of tsetse and of Triatominae essentially restrict their ability to respond to the changing circumstances imposed by available control interventions [40]. This cannot be said of many other vectors, and should be a key factor heralding the long hoped for success.

## Competing interests

The authors declare that they have no competing interests.

## Authors' contributions

CJS and JPK contributed equally to this review.
